# Large cell neuroendocrine carcinoma of the kidney with cardiac metastasis: a case report

**DOI:** 10.1186/s13256-017-1460-7

**Published:** 2017-10-20

**Authors:** Moeka Shimbori, Kimito Osaka, Takashi Kawahara, Ryo Kasahara, Sayuki Kawabata, Kazuhide Makiyama, Keiichi Kondo, Noboru Nakaigawa, Shoji Yamanaka, Masahiro Yao

**Affiliations:** 1 Department of Urology, Yokohama City University Graduate School of Medicine, 3-9, Fukuura, Kanazawa-ku, Yokohama, Kanagawa 2360004 Japan; 2 Departments of Urology and Renal Transplantation, Yokohama City University Medical Center, Yokohama, Japan; 3 Departments of Anatomy and Clinical Pathology, Yokohama City University Hospital, Yokohama, Japan

**Keywords:** Large cell neuroendocrine carcinoma, Kidney, Nephrectomy, Cardiac metastases, Chemotherapy

## Abstract

**Background:**

Primary large cell neuroendocrine carcinoma of the kidney is a rare and generally very aggressive disease. We present a case of a patient with primary large cell neuroendocrine carcinoma of the kidney with cardiac metastasis.

**Case presentation:**

A 59-year-old Japanese man presented to his previous physician with hematuria. Computed tomography revealed masses in the heart and right kidney, and fluorodeoxyglucose-positron emission tomography showed abnormal uptake in the heart. A cardiac biopsy under transesophageal echocardiographic guidance revealed a metastatic tumor. Subsequently, multiple lung lesions were detected, and a right nephrectomy was performed after these metastases were suspected to have originated from renal carcinoma. Large cell neuroendocrine carcinoma of the kidney was ultimately diagnosed. Pancreatic metastasis was detected on computed tomography postoperatively. Three courses of chemotherapy with carboplatin and irinotecan were administered, and were temporarily effective against the metastatic lesions in the lungs and pancreas. However, our patient’s general condition deteriorated with the progression of the lesions, and he died 9 months after his initial examination.

**Conclusions:**

Multi-agent chemotherapy, including platinum-based drugs was effective against large cell neuroendocrine carcinoma metastases, albeit only temporarily. This is the first reported case of large cell neuroendocrine carcinoma with cardiac metastasis.

## Background

Neuroendocrine tumors of the urinary tract are categorized into small cell neuroendocrine carcinomas (SCNEC), large cell neuroendocrine carcinomas (LCNEC), well-differentiated neuroendocrine tumors, and paragangliomas according to the World Health Organization classification of 2016 [1].

Primary LCNEC of the urinary tract is an extremely rare and aggressive disease, and reports of LCNEC of the kidney are exceedingly scarce [2–6]. To the best of our knowledge, there are no reports available on LCNEC with cardiac metastasis. Herein, we report the first case of a patient with LCNEC with cardiac metastasis.

## Case presentation

**Fig. 1:**
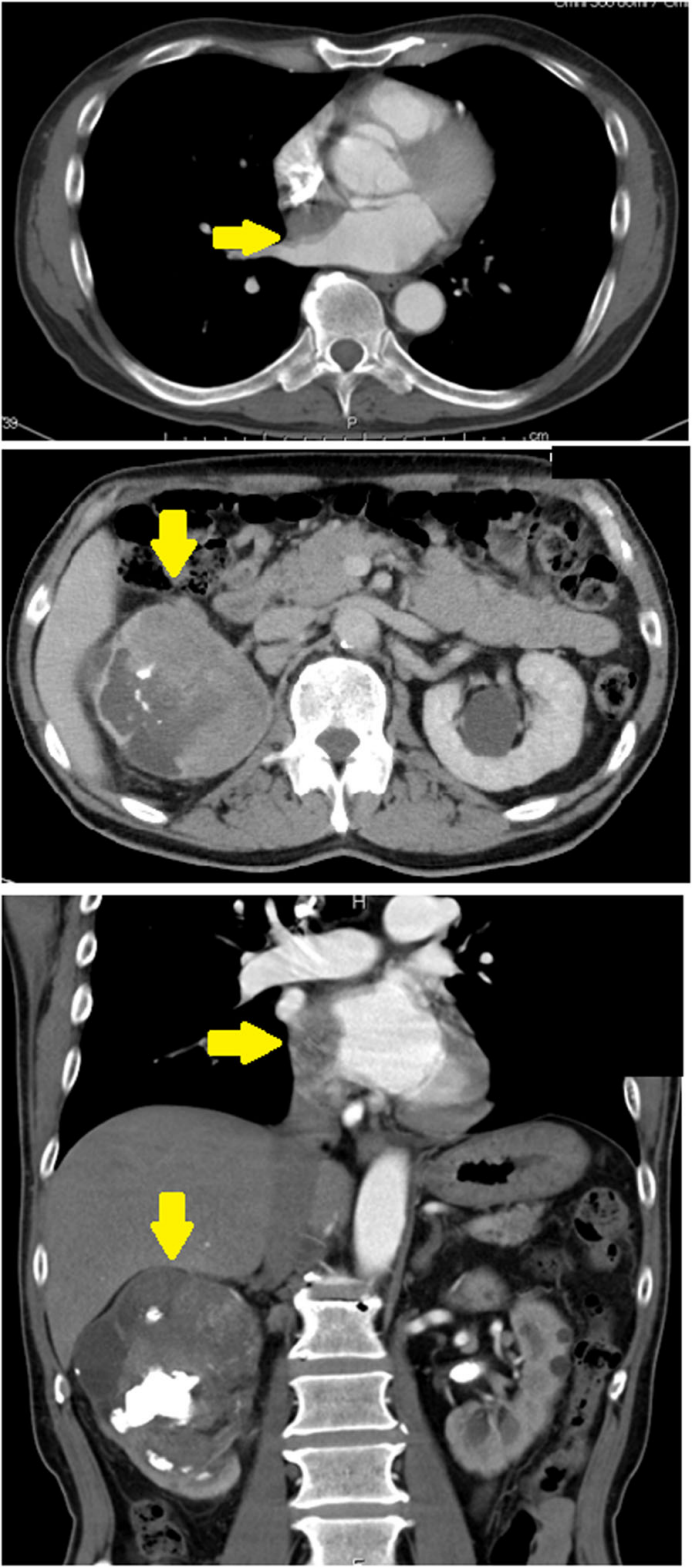
Computed tomography at the first examination. *Top*: computed tomography revealed a 32 mm hypodense mass (*yellow arrow* →) between the left and right atria. *Middle*: computed tomography showed an 86 mm hypodense mass (*yellow arrow*↓) located in the right kidney and extending into the right renal pelvis. *Bottom*: both cardiac (*yellow arrow*→) and renal masses (*yellow arrow*↓) are visible

**Fig. 2:**
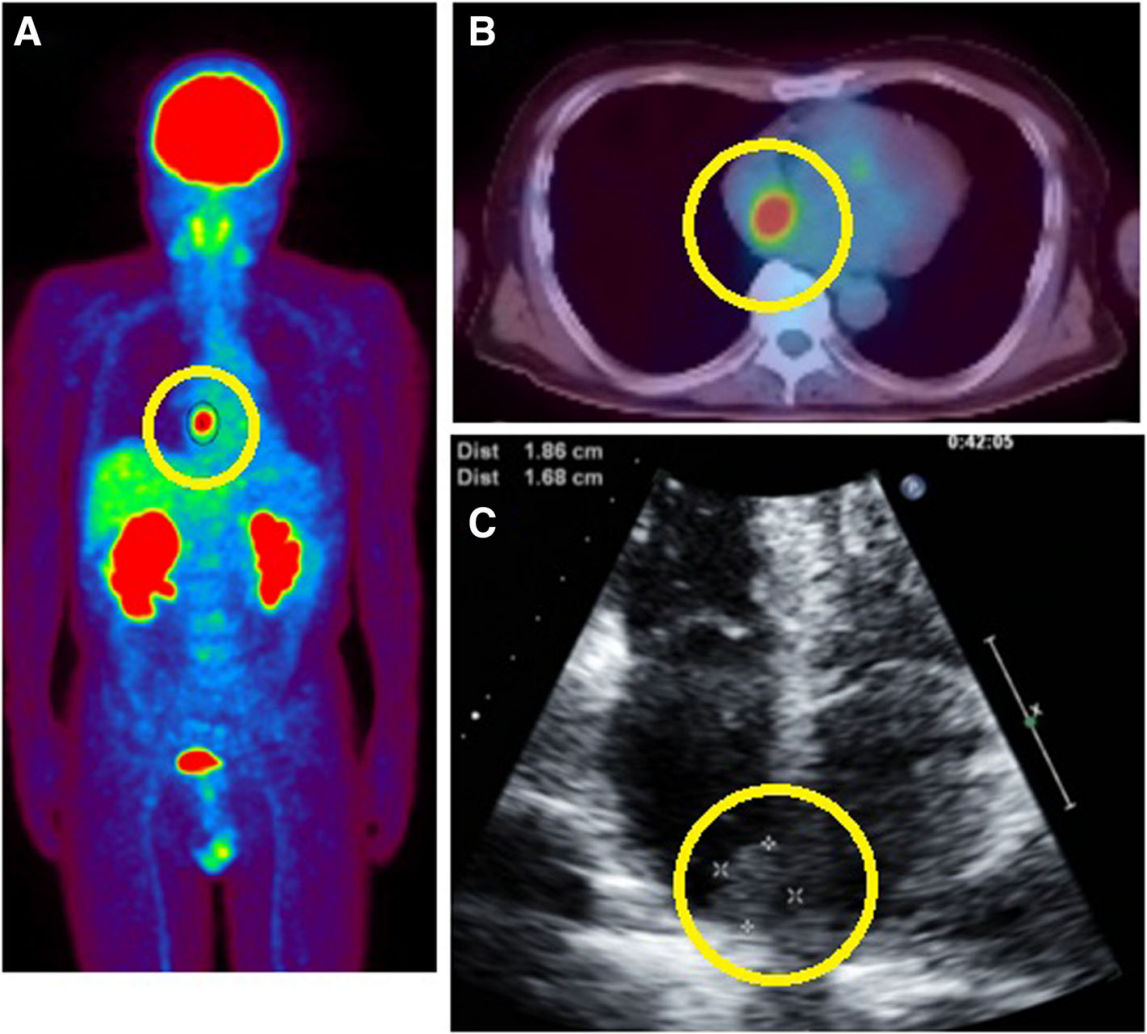
Positron emission tomography-computed tomography. **a** and **b**: Fluorodeoxyglucose uptake in the heart (*circle*), with a maximum standard uptake value (SUVmax) of 9.4. **c**: Echocardiography revealed a 16.8×18.6 mm ill-defined, interatrial septal mass (*circle*) protruding into the right atrium

A 59-year-old Japanese man presented to his previous physician with asymptomatic gross hematuria in July 2014. The patient had no back pain and felt no fatigue. He was a smoker, and had a history of hypertension, renal calculi, and myocardial infarction. He had no personal or family history of malignant tumors, and had no past psychosocial problems. His vital signs and physical and neurological examinations were normal. His laboratory data was notable for anemia (hemoglobin 11.7 g/dL) and decreased renal function (creatinine 1.46 mg/dL). Urinalysis showed hematopyuria (red blood cell ≧50/high power field and white blood cell ≧50/high power field). Computed tomography (CT) revealed bilateral renal stones and an 86 mm hypodense mass located in the right kidney (Fig. 1), as well as a 32 mm hypodense, ill-defined mass in the interatrial septum. Urine cytology was negative, and cystoscopy revealed no bladder lesions. Fluorodeoxyglucose-positron emission tomography (FDG-PET) showed uptake in the heart, with a maximum standardized uptake value (SUVmax) of 9.4 (Fig. 2a and b); the patient was therefore transferred to our hospital in December 2014. Echocardiography revealed an 18.6 mm ill-defined, interatrial septal mass protruding into the right atrium (Fig. 2c). Bone scintigraphy showed no evidence of metastasis. A biopsy was performed under transesophageal echocardiographic guidance to diagnose the cardiac mass; the histopathological diagnosis was metastatic carcinoma. A post-biopsy CT scan revealed multiple new lung metastases. In January 2015, we performed open right radical nephrectomy with concomitant adrenalectomy.

**Fig. 3:**
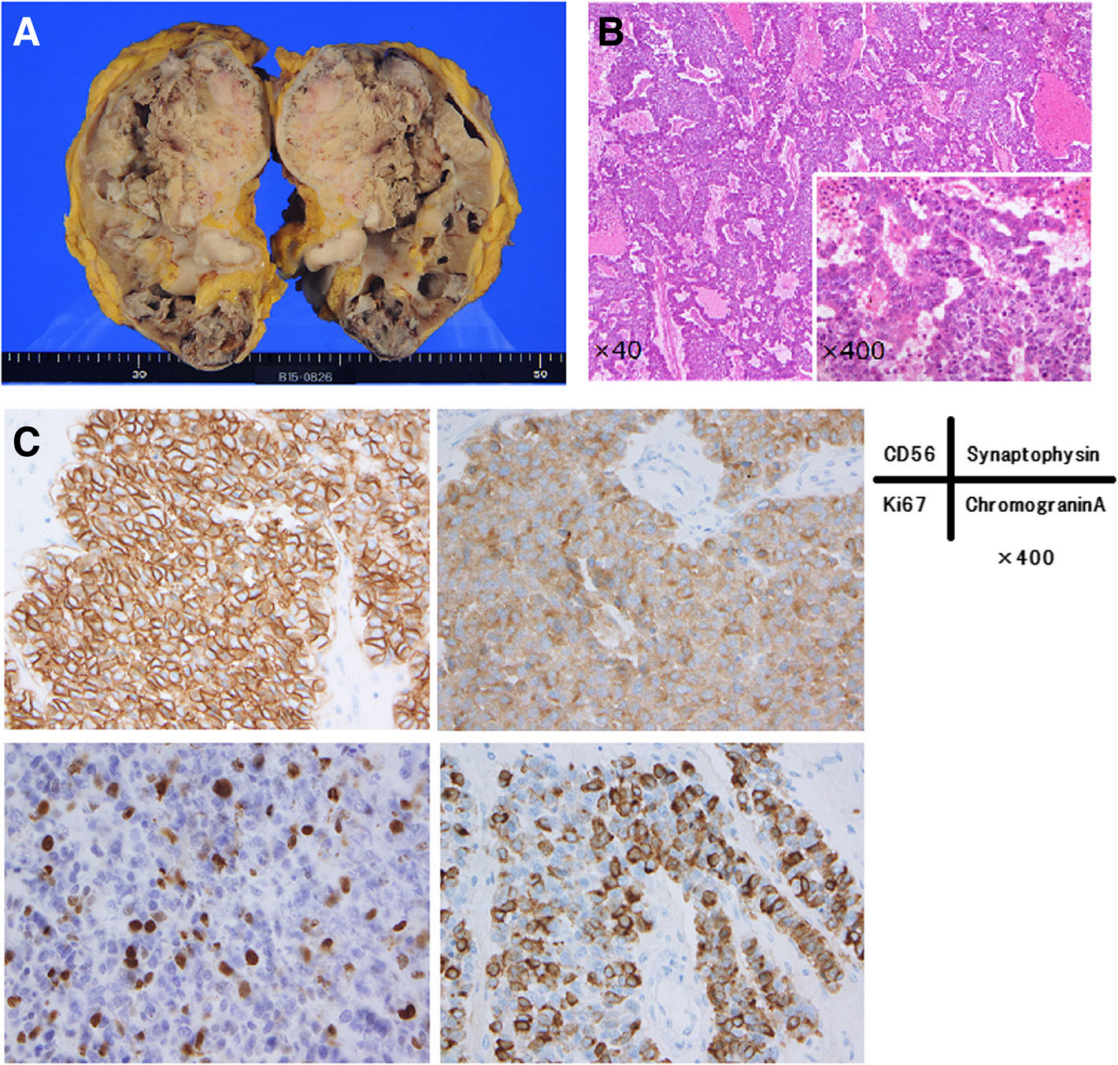
Histopathological examination. **a** Gross picture of the specimen. The tumor was 116×56×19 mm in diameter and occupied most of the renal pelvis. **b** Hematoxylin-eosin staining showed that the tumor was composed of solid nests of tumor cells (and partly of tumor cells cords) with a high nucleus-cytoplasm ratio and central necrosis. **c** Immunohistochemical staining showed that almost all tumor cells were positive for CD56, synaptophysin, and chromogranin. Ki67 was detected in 15–20% of the tumor cells. These features were consistent with neuroendocrine carcinoma

The tumor, which was 116×56×19 mm in size, was vastly disseminated throughout the renal pelvis, and extended into the perinephric fat. A histopathological examination using hematoxylin-eosin staining showed that the tumor was composed of solid nests of malignant cells with a high nuclear/cytoplasmic ratio and central necrosis. Immunohistochemical staining showed that almost all tumor cells were positive for CD56, synaptophysin, and chromogranin A. Ki67 was detected in 15–20% of the tumor cells. These findings led to a diagnosis of right kidney, high grade, pT4N1 LCNEC (Fig. 3). Based on this diagnosis, CD56, synaptophysin, and chromogranin A staining was also performed, on the biopsy specimens from the cardiac mass, which were found to be positive for these markers.

In March 2015, a 22 mm pancreatic tumor was found on CT after surgery.

Our patient was administered three courses of carboplatin (CBDCA) and irinotecan (CPT-11) starting in April 2015, based on postoperative adjuvant chemotherapy protocols for high-grade neuroendocrine carcinomas of the lung. The pancreatic metastasis disappeared, and the multiple lung metastases shrank at a rate of 35%, indicating a partial response (PR). Echocardiography showed stable disease of the cardiac metastasis. The side effects of chemotherapy included diarrhea (Common Terminal Criteria for Adverse Events, CTCAE, grade 2), anorexia (CTCAE grade 3), and neutropenia (CTCAE grade 4). Our patient's general condition deteriorated to an Eastern Cooperative Oncology Group performance status of 2, which point the chemotherapy was discontinued.

**Fig. 4:**
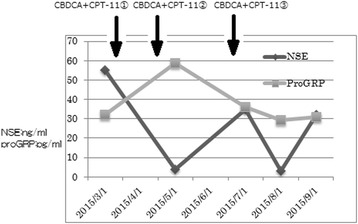
Changes in tumor marker levels. The levels of neuron-specific enolase and pro-gastrin-releasing peptide before chemotherapy were 4.0 ng/mL and 58.5 pg/mL, respectively. Neuron-specific enolase decreased in response to treatment, and increased after treatment discontinuation. Changes in pro-gastrin-releasing peptide did not correlate with treatment. Neuron-specific enolase and pro-gastrin-releasing peptide levels are expressed as ng/mL and pg/mL, respectively

Our patient had elevated tumor marker levels before chemotherapy: with 4.0 ng/mL of neuron-specific enolase (NSE) and 58.5 pg/mL of pro-gastrin-releasing peptide (proGRP). NSE decreased in response to treatment, but increased after discontinuation of treatment. ProGRP increased slightly after treatment, but its change pattern was generally not correlative with treatment (Fig. 4).

In July 2015, bone scintigraphy revealed multiple bone metastases. Our patient's general condition continued to gradually deteriorate, and he died 9 months after diagnosis.

## Discussion

LCNEC of the kidney is extremely rare [2–6]. While various treatments have been attempted, no standard treatment for this disease exists. The outcomes of lung LCNEC is very poor, and its response rate to cisplatin-based chemotherapy is comparable to that of SCNEC [7]. Akamatsu *et al*. reported that surgical resection and chemotherapy were effective for bladder LCNEC [8]. Coelho *et al*. reported that a surgical approach and chemotherapy with platinum were effective for LCNEC of the bladder [9]. In our case, multi-agent chemotherapy, including with platinum-containing drugs, was temporarily effective against metastases arising from LCNEC of the kidney.

As our patient had multiple pulmonary and pancreatic metastases and decreased renal function, the platinum-based anticancer drug CBDCA was selected to protect renal function, and chemotherapy using this agent plus CPT-11 was administered. After two courses of chemotherapy, the pulmonary and pancreatic metastases showed a PR while the cardiac metastasis remained stable, indicating that the efficacy of chemotherapy varies according to the location of the lesions.

Cardiac metastases are considered rare; Bussani *et al*. reported that the incidences of such metastases range from 2.3% to 18.3% in autopsy studies, and are particularly high in malignant pulmonary mesotheliomas, malignant melanomas, pulmonary adenocarcinomas, and breast cancers [10]. In the field of urology, the incidence of cardiac metastases arising from renal carcinoma is relatively high at 7.3% [10]. Many cardiac metastases are pericardial or epicardial; myocardial metastases, as in the present case, are less frequent [10, 11].

In our patient, FDG-PET was performed to examine the renal tumor, whereupon abnormal uptake in the heart led to the detection of cardiac tumors. As 90% of cardiac metastases from malignant tumors are asymptomatic [10], they are rarely detected before death. However, a previous study by Sato *et al*. found that FDG-PET is a useful modality for the detection of cardiac tumors [12].

Cardiac metastases are rarely limited to the heart itself, and the development of tachycardia, arrhythmias, cardiomegaly, or heart failure in a patient with carcinoma should raise the suspicion of cardiac metastases [11]. However, intramyocardial masses, as detected in the present case, are usually clinically silent; our patient did not experience subjective or objective symptoms [11].

Cardiac tumors are usually treated with surgical resection. In contrast, metastatic cardiac tumors are associated with a poor prognosis because no specific treatment has been established for such lesions: as such, they are often lumped with other metastases in multiple organs [13].

Although not definitively diagnosed pathologically, the cardiac tumor in our case was clinically judged to be a cardiac metastasis of LCNEC of the kidney, because the pathological findings indicated metastatic carcinoma and were consistent with LCNEC of the kidney. Furthermore a workup revealed no evidence of malignant tumors at other sites. As the patient’s family did not consent to an autopsy, we were unable to make a histopathological diagnosis of the cardiac metastasis.

Neuroendocrine carcinoma of the kidney is a rare disease associated with a very poor prognosis for which no systematic therapy exists. It is important to control lesions using multimodal therapy that includes surgery, chemotherapy, and radiotherapy. Cardiac metastasis, which is difficult to treat, was already detected during the patient’s initial examination, and multiple organ metastases then progressed rapidly.

## Conclusions

We encountered a rare case of a patient with primary LCNEC of the kidney with cardiac metastasis. Our patient was treated with surgery for pathological diagnosis, followed by combination chemotherapy with CBDCA and CPT-11. Although the treatment had limited efficacy, levels of the tumor markers NSE and proGRP decreased, and the tumor shrank in response chemotherapeutic agents.

## Abbreviations

CBDCA: Cyclobutane dicarboxylate platinum
CPT11: Camptothecin 11
CT: Computed tomography
CTCAE: Common Terminal Criteria for Adverse Events
FDG-PET: Fluorodeoxyglucose-positron emission tomography
LCNEC: Large cell neuroendocrine carcinoma
NSE: Neuron-specific enolase
PR: Partial response
ProGRP: Pro-gastrin-releasing peptide
SCNEC: Small cell neuroendocrine carcinoma
SUV: Standardized uptake value
